# Determination of related factors about diagnostic and treatment delays in patients with smear-positive pulmonary tuberculosis in Turkey

**DOI:** 10.3906/sag-2001-89

**Published:** 2020-08-26

**Authors:** Mustafa Hamidullah TÜRKKANI, Tarkan ÖZDEMİR, Çiğdem ÖZDİLEKCAN

**Affiliations:** 1 Department of Chest Diseases, Dr. Nafiz Körez Sincan State Hospital, Ankara Turkey; 2 Department of Chest Diseases, Dr. Abdurrahman Yurtaslan Ankara Oncology Training and Research Hospital,University of Health Sciences, Ankara Turkey

**Keywords:** Health policy, tuberculosis control, patient delay, diagnosis delay

## Abstract

**Background/aim:**

This study aimed to analyze delays in diagnosis and treatment by defining the related demographic and clinical factors, to reveal obstacles, and to develop essential attempts to help reduce treatment delays.

**Materials and methods:**

We created a questionnaire on the subject of delays in diagnosis and treatment in tuberculosis (TB) control to be administered to the patients. The forms were distributed to dispensaries across the country by the General Directorate of Public Health via an official letter.

**Results:**

The study included 853 new patients with smear-positive pulmonary TB. The mean patient delay was 18.06 ± 22.27 days, the mean diagnosis delay was 35.63 ± 34.86 days, and the mean treatment delay was 0.90 ± 2.39 days. We found no association between sex, age, literacy, residential location, the presence of chronic respiratory diseases, and patient delay. It was determined that patient delay was shorter for patients with hemoptysis, fever, dyspnoea, and chest pain. In women, the diagnosis delay was longer than in men.

**Conclusion:**

In the diagnosis process of patients with tuberculosis, it was determined that there was an improvement in the patient delay; however, the improvement in the diagnosis delay was still not acceptable as an ideal duration.

## 1. Introduction

Early diagnosis, administering treatment with a standard regimen, and starting treatment immediately are some of the primary targets for the elimination of tuberculosis (TB) [1–3]. Within the context of prevention, the End TB Strategy suggests a patient-centered care to focus on early diagnosis, treatment, and prevention [4]. Delays in the diagnosis and treatment of pulmonary TB are a great obstacle in the elimination of TB globally [5].

Delays in diagnosis and treatment increase the rate of contamination [6]. Contamination most probably happens before the effective treatment of the index cases [7]. With treatment, the frequency of coughing of the patient with TB and the number of bacteria in their sputum decreases rapidly, which indicates both symptomatic relief and decreased contamination [8,9]. With effective treatment, the contamination feature of TB is diminished practically within 2–3 weeks [2].

Delays in diagnosis and treatment lead to increasing complications and long-term periods of contagiousness in public, which causes higher amounts of debts and increase in mortality [10]. The evaluation of delays in diagnosis is important for interpreting the control activity of TB and defining the obstacles [11]. Delays between the onset of TB symptoms and the initiation of the treatment are caused either by the patient, healthcare system, or a combination of both. Delays in diagnosis are usually attributed to healthcare systems [12].

Turkey has passed through a health transformation [13] and this transformation has had positive effects on the control of TB [14]. This will be the first study that almost entirely consists of a Turkish database. We aimed to analyze delays in diagnosis and treatment by defining the related demographic and clinical factors, to reveal obstacles, and to develop essential attempts to help reduce treatment delays.

## 2. Methods

### 2.1 National TB control 

The planning and administration of the TB control program in Turkey are under the responsibility of the TB departments affiliated with the General Directorate of Public Health within the Ministry of Health of the Republic of Turkey. These departments are currently planning and carrying out TB control work within tuberculosis dispensaries with the coordination of the Provincial Health Directorates, family physicians, hospitals, and all institutions of health. 

In Turkey, as a healthcare service, all diagnostic and treatment procedures of TB are maintained without charge. The institutions of the Ministry of Health provide all the necessary medications without charge for the patients with TB and the individuals with a history of contact with TB bacilli, including cases that are multi drug-resistant, without considering the social insurance status of the patient.

In Turkey’s TB control program, the endpoint units are the TB dispensaries. Patients who are diagnosed as having TB are referred to the dispensaries, which are responsible for duties including diagnosis, treatment, patient follow-ups, notifying patients, registration, keeping statistics, immunization, scanning, supplying medication, education, information activities, coordination, and consultation services. 

### 2.2. Study design

We planned this study for the notification of patients with TB based on the delay in diagnosis and treatment and to determine the obstacles. We created a questionnaire on the subject of delays in diagnosis and treatment in TB control to be administered to the patients. The form basically tries to obtain information about the patients and the delays in their diagnosis and treatment. The questions included the general demographic characteristics of the patients, major symptoms, the date of therapy initiation, the date of presentation to a healthcare institute, the first admission to the medical department, the date of presentation when the TB diagnosis was made, the medical department of the physician who first diagnosed TB, and the date of the initiation of the treatment. We received the written consent of the General Directorate of Public Health in order to provide and use the data.

### 2.3. Data collection

The questionnaires were distributed to dispensaries across the country by the General Directorate of Public Health via an official letter. The data were collected from 157 dispensaries located in 81 provinces. The questionnaires were administered by the physicians of the dispensaries to patients with smear-positive pulmonary TB who volunteered to complete the questionnaire. The patients were diagnosed between January 1 and December 31 2018 and were registered to the dispensaries. Before completing the questionnaire, all the patients provided their written informed consent. The patients were enlightened regarding any further questions by the physicians of the dispensaries. The smear-negative pulmonary TB cases, extrapulmonary TB cases, retreatment TB cases, and the patients under 18 years were not included in the study. 

### 2.4. Definitions

Patient delay: The delay between the symptom onset and the admission of the patient to a healthcare institution (time unit = days);

Diagnosis delay: The delay between the admission to a healthcare institution and receiving a diagnosis (time unit = days);

Treatment delay: The delay between the diagnosis and the initiation of the treatment (time unit = days);

Total delay: The delay between the symptom onset and the treatment administration (time unit = days).

### 2.5. Statistics

Statistical analyses were performed using the SPSS software version 18 (SPSS Inc., Chicago, IL, USA). The normality of distribution of the variables was evaluated using visual (histograms and probability graphics) and analytical methods (Kolmogorov–Smirnov/Shapiro–Wilk tests). The time frames of the first examination admission and the diagnosis were compared within various groups according to symptoms, the institution, and the diagnosing physician using the Kruskal–Wallis test because these parameters were not normally distributed. Comparisons between the two variables were performed using the Mann-Whitney U test and evaluated using the Bonferroni correction. For variables in which at least one was not normally distributed or ordinal, the correlation coefficient and statistical significance were calculated using the Spearman test. In the analysis, a type 1 error rate of 5% was used for possible statistically significant factors .

## 3. Results

This nationwide study involved 853 new patients with smear-positive pulmonary TB. The age range of the patients was 58.05 ± 17.85 [median = 60 (range 21–96)] years. Among the participants, 581 (68.1%) were male, and 31.9% (n = 272) were female. As for the place of residence, 170 (20%) of the patients were living in villages. The literacy rate was 85.2% (n = 727) among the participants and 14.8% (n = 126) were illiterate. Moreover, 158 patients (18.5%) had a history of chronic pulmonary disease. When it comes to smoking habits, it was seen that 413 patients (48.4%) were current smokers, 38.3% (n = 327) were never smokers, and the remaining 13.3% (n = 113) were former smokers. The most common initial symptom was cough (n = 509, 59.7%). The physicians who initially admitted the patients were from the departments of chest diseases (n = 340, 39.9%), internal medicine (n = 175, 20.5%), and family physicians (n = 139, 16.3%). We detected the patient delay as 18.06 ± 22.27 [median = 10 (range 0–113)] days, the diagnosis delay as 35.63 ± 34.86 [median = 23 (range 0–151)] days, and the treatment delay as 0.90 ± 2.39 [median = 0 (range 0–14) days]. The majority (88.4%, n = 754) of the patients were diagnosed by a chest diseases physician. Three institution types that had the highest diagnostic rates were university hospitals and education and research hospitals (32.8%, n = 280), chest diseases hospitals (29.3%, n = 250) and government hospitals (22.0%, n = 187), respectively (Table 1).

**Table 1:** General characteristics of the study groups.

Sex n, (%)	Male	581 (68.1)
	Female	272 (31.9)
Residence n, (%)	City center	333 (39)
	Town center	350 (41)
	Village	170 (20)
Education n, (%)	Illiterate	126 (14.8)
	Literate	727 (85.2)
Previous chronic pulmonary disease n, (%)	No	695 (81.5)
	Yes	158 (18.5)
Smoking status n, (%)	Never	327 (38.3)
	Current	413 (48.4)
	Former	113 (13.3)
Initial symptom n, (%)	Cough	509 (59.7)
	Fever	84 (9.8)
	Loss of weight	75 (8.8)
	Night sweating	59 (6.9)
	Chest pain	35 (4.1)
	Fatigue	36 (4.2)
	Hemoptysis	26 (3.0)
	Dyspnoea	22 (2.6)
	Hoarseness	4 (0.5)
	Loss of appetite	3 (0.4)
Initial physician on admission n, (%)	Chest diseases	340 (39.9)
	Internal medicine	175 (20.5)
	Family physician	139 (16.3)
	ER physician	107 (12.5)
	Otorhinolaryngology	36 (4.2)
	Dispensary’s physician	22 (2.6)
	Infectious diseases	19 (2.2)
	Other	15 (1.8)
Diagnosing physician n, (%)	Chest diseases	754 (88.4)
	Dispensary’s physician	63 (7.4)
	Infectious diseases	16 (1.9)
	Internal medicine	13 (1.5)
	Otorhinolaryngology	2 (0.2)
	Primary care physician	2 (0.2)
	Other	3 (0.4)
Institution of diagnosis n, (%)	University—training and research hospital	280 (32.8)
	Chest diseases hospital	250 (29.3)
	Government hospital	187 (22.0)
	Dispensary	78 (9.1)
	Private hospital	58 (6.8)
Time period of delay, mean days ± SD	Delay of the patient	18.06 ± 22.27
	Delay in the diagnosis	35.63 ± 34.86
	Delay in the treatment	0.90 ± 2.39

SD: Standard deviation; ER: Emergency room.

There was no significant correlation between age and the patient delay (r = –0.048, P > 0.05). The patient delay showed no association (P > 0.05) with sex. Furthermore, there was no association between literacy levels and the patient delay (P > 0.05). However, an association was found between smoking and the patient delay (P < 0.001). In current smokers, the patient delay (median = 16 days, n = 413) was longer than in never smokers (median = 11 days, n = 440). No association was found between the presence of chronic respiratory disease (CRD) and the patient delay (P > 0.05). Also, patient delay showed no association according to the residence of the patients (P > 0.05) (Table 2).

**Table 2:** The analysis of parameters that could affect the time period of admission.

Sex	Female		Male				
	Mean ± SD	Med (Min-Max)	Mean ± SD		Med (Min-Max)		P 0.745^m^
Patient delay (days)	23.47 ± 25.38	14 (0–114)	23.34 ± 24.60		14 (0–120)	
Education	Literate		Nonliterate				
	Mean ± SD	Med (Min-Max)	Mean ± SD		Med (Min-Max)		P 0.475^m^
Patient delay (days)	22.99 ± 24.43	14 (0–120)	25.63 ± 27.06		14 (0–117)		
Smoking	Current smoker		Nonsmoker				
	Mean ± SD	Med (Min-Max)	Mean ± SD		Med (Min-Max)		P 0.001^m^
Patient delay (days)	26.31 ± 25.88	16 (0–114)	20.63 ± 23.52		11 (0–120)		
CRD	CRD present		CRD absent				
	Mean ± SD	Med (Min-Max)	Mean ± SD		Med (Min-Max)		P 0.959 ^m^
Patient delay (days)	23.34 ± 25.36	11.5 (0–114)	23.39 ± 24.74		14 (0–120)		
Residence	Centrum		Town center		Village		
	Mean ± SD	Med (Min-Max)	Avg ± SD	Med (Min-Max)	Avg ± SD	Med (Min-Max)	P0.094 ^K^
Patient delay (days)	23.59 ± 23.91	(14–120)	24.38 ± 25.77	(14–113)	20.94 ± 24.65	(10–117)	

CRD: Chronic respiratory disease; SD: Standard deviation; Med: Median; Min: Minimum; Max: Maximum .

**Figure 1:**
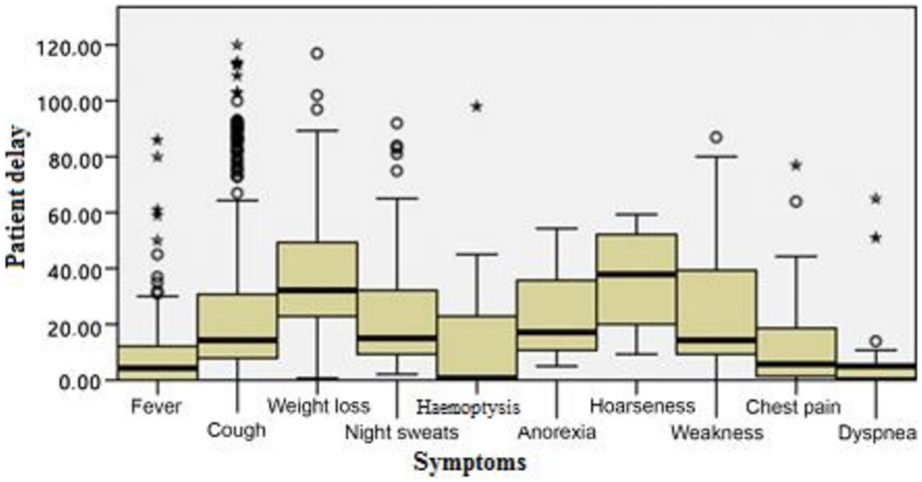
The relationship between the initial symptoms and patient delay: According to the initial symptoms of the patients, the patient delay showed a statistical significance (P < 0.001). It was determined that this difference was caused by patients who had dyspnoea, fever, hemoptysis, and chest pain.

**Figure 2:**
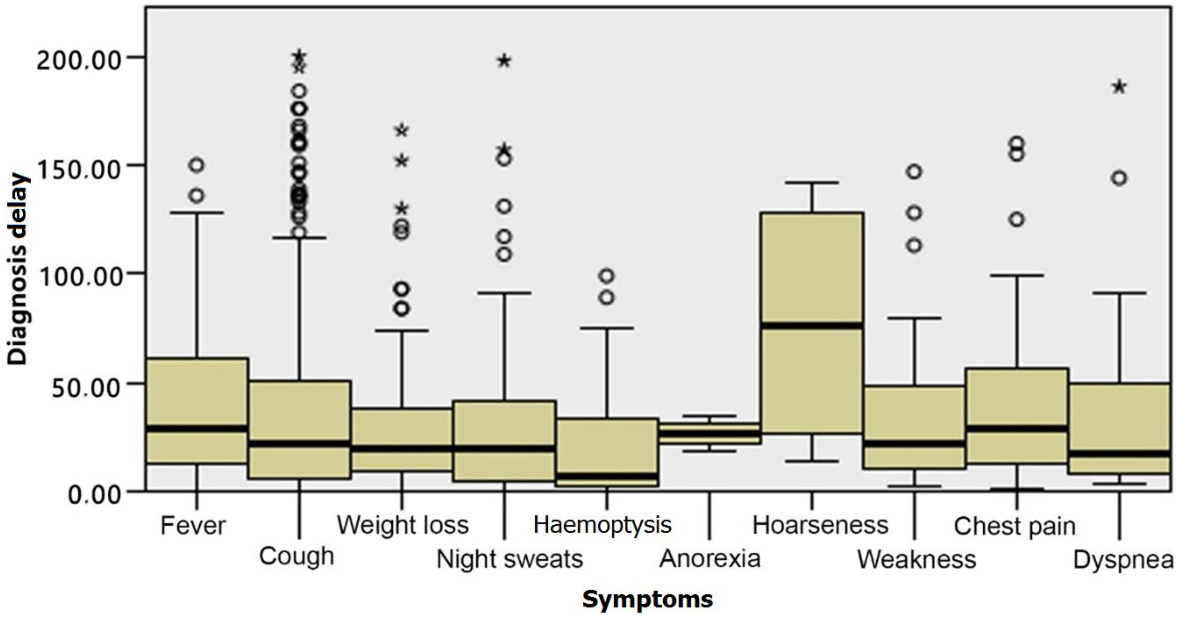
The relationship between the initial symptoms and the diagnosis delay: There was no statistical significance (P > 0.05) between the patients’ first symptoms and the diagnosis delay.

According to the initial symptoms of the patients, the patient delay showed a statistical significance (P < 0.001). Within the multiple comparisons performed using the Mann–Whitney U test, it was determined that this difference was caused by patients who had dyspnoea, fever, hemoptysis, and chest pain. The patient delay of the patients with hemoptysis (median = 1 day), fever (median = 4 days), dyspnoea (median = 5 days), and chest pain (median = 6 days) was significantly shorter compared with the other symptom types (P < 0.001). On the other hand, there was no statistical significance between the patients’ first symptoms and the diagnosis delay (P > 0.05) (Figures 1 and 2).

Between the factors of age and the diagnosis delay, a low degree of correlation was present (r = 0.102, P = 0.003). The diagnosis delay in women (median = 27 days, n = 272) was found to be longer than in men (median = 19 days, n = 581) (P < 0.001). No significance was found between the diagnosis delay and the CRD status (P > 0.05). In current smokers (median = 19 days, n = 413), the diagnosis delay was found to be shorter than in never smokers (median = 26 days, n = 440) (P < 0.001). There was a moderate degree of correlation between the total number of consulted physicians and the diagnosis delay (r = 0.460, P = 0.001). There was a statistically significant correlation between the total number of examinations and the diagnosis delay (r = 0.515, P = 0.001) (Table 3).

**Table 3:** The analysis of the parameters that could affect the time period of diagnosis.

Sex	Female		Male		
	Mean ± SD	Med (Min-Max)	Mean ± SD	Med (Min-Max)	P< 0.001^m^
Diagnosis delay (days)	40.5 ± 39.74	27 (0–195)	32.1 ± 36.79	19 (0–200)
Smoking	Current Smoker		Non-Smoker		
	Mean ± SD	Med (Min-Max)	Mean ± SD	Med (Min-Max)	P 0.001^m^
Diagnosis delay (days)	30.73 ± 35.81	19 (0–198)	38.6 ± 39.49	26 (0–200)	
CRD	CRD present		CRD absent		
	Mean ± SD	Med (Min-Max)	Mean ± SD	Med (Min-Max)	P 0.205^m^
Diagnosis delay (days)	35.80 ± 35.99	26 (1–168)	34.52 ± 38.38	21 (0–200)	

CRD: Chronic respiratory disease; SD: Standard deviation; Med: Median; Min: Minimum; Max: Maximum

There was a significant difference between the patients’ status of initial admission to a physician and the diagnosis delay (P < 0.001). According to the Mann–Whitney U test used for multiple comparisons, it was found that this difference was caused by the patient group that was admitted by physicians from dispensaries, chest diseases departments, and emergency room (ER) wards. The diagnosis delay was significantly shorter in patients who were admitted by dispensary physicians (median = 4.5 days), chest diseases specialists (median = 11 days), and ER ward physicians (median = 16 days) than other specialists (Table 4).

**Table 4:** Analysis of the relationship between the first physician and diagnosis delay.

Physician for diagnosis	n	Avg ± SD (days)	Med (days)	(Min-Max) (days)	P
Dispensary physician	22	10.05 ± 19.11	4.5	0–92	< 0.001*
Chest diseases	340	36.10 ± 27.24	11	0–200	
ER physician	107	30.41 ± 28.17	16	0–155	
Infectious diseases	19	30.63 ± 33.38	22	5–150	
Internal medicine	175	42.91 ± 39.26	30	1–195	
Primary care physician	139	44.60 ± 37.73	31	2–176	
Otorhinolaryngology	36	58.48 ± 49.70	41	1–184	
Other	15	50.47 ± 38.56	41	10–157	

SD: Standard deviation; ER: Emergency room; Med: Median; Min: Minimum; Max: Maximum. *Dispensary, chest, and ER physicians were found to have statistically significant differences in terms of diagnosis time when compared with other physicians (P < 0.001).

After admission to healthcare institutions, statistically significant differences were found between diagnosis periods (P < 0.001). As a result of multiple comparisons performed using the Mann–Whitney U test, the diagnosis period after admission to university training and research hospitals were found to be significantly longer (median = 6 days) when compared with other institutions (P < 0.001) (Table 5).

**Table 5:** The comparison of the time period of diagnosis between the institutions that performed accurate diagnosis of TB.

Institution of diagnosis	n	Mean ± SD (days)	Med (days)	(Min-Max) (days)	P
University–training and research hospital	280	14.46 ± 21.59	6	0–161	< 0.001*
Chest diseases hospital	250	9.19 ± 16.30	3	0–117	
Government hospital	187	10.79 ± 18.83	4	0–128	
Dispensary	78	5.41 ± 10.99	3	0–92	
Private hospital	58	9.86 ± 13.01	4.5	0–64	

SD: Standard deviation; Med: Median; Min: Minimum; Max: Maximum. * University–training and research hospitals were found to have statistically significant differences in terms of diagnosis period when compared with other hospitals (P < 0.00).

## 4. Discussion

Our study, which was performed regarding the delays in the diagnosis and treatment of TB, is the first one to include major data of Turkey. In our study, the patient delay was 18.06 ± 22.27 days, the diagnosis delay was 35.63 ± 34.86 days, and the treatment delay was 0.90 ± 2.39 days. We found no associations between the patient delay and sex, age, literacy, residential location, and the presence of CRD. However, there was an association between smoking status and the patient delay. It was determined that the patient delay was shorter in patients with hemoptysis, fever, dyspnoea, and chest pain. Also, the patient delay was longer in women than in men.

In Turkey, studies concerning delays in diagnosis and treatment are usually based on hospitals or clinics. In a retrospective study conducted in a university hospital in Ankara between 1994 and 1997, which was based on the time period between the symptom onset and treatment in patients with TB, it was determined that the median days was 62, based on 28 patients who were smear-positive for TB [15]. In a study carried out with 81 patients who were TB smear-positive and hospitalized in 1998 and had started their treatment in a training and research hospital in Ankara, which is a reference center for TB, it was determined that the days of the patient delay was 82.6 ± 70.7, the physician delay was 41.4 ± 55.8, and the total delay was 124.0 ± 113.7 days [16]. In a study performed in 1999 in the chest diseases department of a military hospital in Istanbul, it was determined that the total delay in diagnosis in all the patients was found to be a mean 16.3 days, (median = 21 days) [17]. According to a study conducted in 1999 with 134 patients hospitalized for smear-positive pulmonary TB in a training and research hospital in Istanbul, which was a reference center for TB, the mean days of admission for the patients were 26.9, the referral was 9.2, the time period for diagnosis was 3.9, and the initiation of the treatment was 1.3 days. The median time periods were 17.5, 3.5, and 3.1 days, respectively [18]. In another study carried out in the same hospital in 2001 with 204 hospitalized patients with smear-positive pulmonary TB, it was determined that the mean days of admission were 31.4, the referral was 22.1 days, diagnosis was 3.3 days, and the initiation of the treatment was 1.4 days. The median time periods were 17.5, 11.0, 1.5, and 1.0 days, respectively [19]. Later, a new study was carried out in the same hospital in 2010 with 136 new patients hospitalized with pulmonary TB. In this study, the mean and median days between the initiation of symptoms and the initiation of treatment were 64.7 and 48 in smear-positive cases (n = 71) and 99.8 and 61 days in smear-negative cases (n = 65) [20]. In another reference hospital for TB in Istanbul, a study performed in 2004 revealed that out of 151 new patients with smear-positive pulmonary TB, the mean days between the symptom onset and the first visit to a physician was 46.4, the delay of referral was 28.9 days, the delay of diagnosis was 2.4 days, and the delay of treatment initiation was 0.8 days [21]. Based on national parameters, it was determined that in Turkey the patient delay, the diagnosis delay, and the treatment delay had decreased. However, the diagnosis delay, which was found to be 35 days, cannot be accepted as an ideal duration. Among all disciplines, the knowledge and awareness of TB diagnosis could be an underestimated issue. Therefore, patients admitted with pulmonary symptoms are nearly always evaluated with a suspicion of TB.

In a review study in which studies from 78 countries were evaluated and systematically compiled, the patient delay was found as 81 days, the physician delay was 29.5 days, and the treatment delay was 7.9 days [5]. The diagnosis delay was still an obstacle in TB prevention and control programs in low- and medium-income levels [11]. Health illiteracy, poverty, and other personal reasons cause delays in admission to healthcare institutions, and consequently, delays in the diagnosis of TB. Inadequate health systems also cause delays in diagnosis and treatment. The health system in Turkey has been through a period of transformation [13] and one of the positive outcomes of this transformation in TB control is the shorter time delays in diagnosis and treatment. 

The most frequent reason for the patient delay is that patients with TB tend to neglect the symptoms [22]. In a previous study performed in Italy, one of the most frequent reasons for delays in treatment was the light nature of the symptoms (82%) and feeling healthy (76%) [23]. In our study, we found that patients whose first symptoms were cough, weight loss, night sweats, exhaustion, and hoarseness tended to neglect these symptoms and consult a physician much later. We found an association between the first symptoms of patients and the patient delay. However, we found no association between the first symptoms and the diagnosis delay.

There were no associations between the patient delay and sex in our study. We found that the diagnosis delay was longer in women (median = 27 days) than in men (median = 19 days). In a study performed in England, the diagnostic delay was found to be associated with being female [24]. In China, female sex was related with a risk of diagnostic delay [25,26].

Unlike most studies, we found no relation between the patient delay and age, literacy, and the place of residence. The patient delay was longer in smokers (median = 16 days) than in non-smokers (median = 11 days). Thus, in current smokers, the diagnosis delay was shorter (median = 19 days) than in non-smokers (median = 26 days). In a study conducted in Serbia, a medium, positive, and statistically significant correlation was found between the number of cigarette smoked and the patient delay [27]. 

We determined that there was a medium correlation between the total number of consultant physicians and the delay in the diagnosis, but there was a higher correlation between the number of examinations and the diagnosis delay. A study performed in India found out that an increased number of consultations and the diagnostic delay were statistically correlated [28]. In a report from Ethiopia, it was emphasized that the systems of initial admission to practitioners were independent indicators of delays in health systems [29]. In a study from Switzerland, one of the main indicators of delays in the health system was evaluated by at least 3 or 4 physicians before initiating the treatment [30]. 

We found an association between the patients’ first visit to a physician and the diagnosis delay. We determined that the diagnosis delay was significantly shorter in patients who were consulted by chest physicians, dispensary physicians, and ER ward physicians than by other physicians. In a study performed in Italy, it was observed that the delay was longer when patients were first evaluated by family physicians [23]. According to a study performed in Ethiopia, seeing a family physician first was one of the independent indicators of the health system [29].

Efforts should be focused on finding patients using the existing systems and developing new strategies to improve patient care-seeking behaviors [11]. We think that strategies regarding the patient delay are vital for the success of TB control programs. We care about raising the awareness of TB, both in the public and among physicians. We conducted the first study to analyze the delays in diagnosis and treatment around Turkey and determined that the Turkish healthcare system has improved its status in these regards. We advise improving the processes in order to improve the awareness of patients about the symptoms of TB and to increase health literacy. 

The limitation of our study can be considered as the volunteer-based completion of the questionnaires by the participants. Thus, this situation does not cover all the patients who were smear-positive in 2018.

In this study covering new patients with smear-positive TB, no significant relation was found between the patient delay and age, sex, education, residential location, and the presence of CRD. However, the patient delay was longer in current smokers than in never smokers. Also, the patient delay was less in patients with symptoms of hemoptysis, fever, and dyspnoea, and chest pain. The diagnosis delay was significantly shorter in patients who were admitted by dispensary physicians, chest diseases specialists, and ER ward physicians than by other specialists. The diagnosis delay was longer in women than in men. In the diagnostic process of patients with TB, it was determined that there was an improvement in the patient delay; however, the diagnosis delay was still not acceptable as an ideal duration.

## Acknowledgement/Disclaimers/Conflict of interest

We would like to thank the dispensary healthcare providers, Dr. Erhan Kabasakal, Dr. Ayşegül Yıldırım, Funda Baykal, and Zehra Yıldırım for their contribution and support for this study.

None of the authors have any conflict of interest. The authors declare that no funding has been received to carry out this study and/or to prepare this manuscript.

## Informed consent

All participants provided their informed consent. We obtained the written consent of the General Directorate of Public Health in order to collect and use the data (60949272-134.99 / 01.19.2018).

## References

[ref1] (2010). World Health Organization. The Global Plan to Stop TB 2011-2015: Transforming the fight towards elimination of tuberculosis. Geneva, Switzerland: World Health Organization.

[ref2] (2011). Republic of Turkey Ministry of Health. The Guideline of Tuberculosis Diagnosis and Treatment. Ankara, Turkey: Republic of Turkey Ministry of Health.

[ref3] (2008). A systematic review of delay in the diagnosis and treatment of tuberculosis. BMC Public Health.

[ref4] (2014). World Health Organization. The End TB Strategy, Global strategy and targets for tuberculosis prevention, care and control after 2015. The official text approved by the Sixty seventh World Health Assembly. Geneva, Switzerland: World Health Organization.

[ref5] (2019). Empirical evidence of delays in diagnosis and treatment of pulmonary tuberculosis: systematic review and meta-regression analysis. BMC Public Health.

[ref6] (2017). Delays in diagnosis and treatment of pulmonary tuberculosis in AFB smear-negative patients with pneumonia. The International Journal of Tuberculosis and Lung Disease.

[ref7] (1981). Infectiousness of pulmonary tuberculosis after starting chemotherapy: review of the available data on an unresolved question. American Journal of Infection Control.

[ref8] (1969). Cough frequency and infectivitiy in patients with pulmonary tuberculosis. The American Review of Respiratory Disease.

[ref9] (1980). The early bactericidal activity of drugs in patients with pulmonary tuberculosis. The American Review of Respiratory Disease.

[ref10] (2018). Delayed diagnosis of tuberculosis: risk factors and effect on mortality among older adults in Hong Kong. Hong Kong Medical Journal.

[ref11] (2017). Delay in diagnosis of pulmonary tuberculosis in low-and middle-income settings: systematic review and meta-analysis. BMC Pulmonary Medicine.

[ref12] (2005). Diagnostic and treatment delay among pulmonary tuberculosis patients in Ethiopia: a crosssectional study. BMC Infectious Diseases.

[ref13] (2012). Republic of Turkey Ministry of Health. Turkey Health Transformation Program Evaluation Report (2003-2011). Ankara, Turkey: Republic of Turkey Ministry of Health.

[ref14] (2013). Effects of the Health Transformation Programme on tuberculosis burden in Turkey. Respiratory Medicine.

[ref15] (2000). Delay in the initiation of anti-tuberculosis treatment. Tuberculosis and Thorax.

[ref16] (2001). Patient and doctor delay in tuberculosis control. Tuberculosis and Thorax.

[ref17] (2005). Delay in the diagnosis of Turkish servicemen with pulmonary tuberculosis. Military Medicine.

[ref18] (2001). Delays in the diagnosis and treatment of hospitalized patients with smear-positive pulmonary tuberculosis. Respiratory Medicine.

[ref19] (2004). Factors affecting delays in diagnosis and treatment of pulmonary tuberculosis in a tertiary care hospital in İstanbul, Turkey. Medical Science Monitor.

[ref20] (2014). Patient and doctor delays in smear-negative and smear-positive pulmonary tuberculosis patients attending a referral hospital in İstanbul, Turkey. The Scientific World Journal.

[ref21] (2006). Patterns of delays in diagnosis amongst patients with smear - positive pulmonary tuberculosis at a teaching hospital in Turkey. Clinical Microbiology and Infection.

[ref22] (2016). Diagnostic delay among adults with pulmonary tuberculosis in a high gross domestic product per capita country: reasons and magnitude of the problem. International Journal of Preventive Medicine.

[ref23] (2018). Determinants of patient and health care services delays for tuberculosis diagnosis in Italy: a cross-sectional observational study. BMC Infectious Diseases.

[ref24] (2012). Delay from symptom onset to treatment start among tuberculosis patients in England,. Epidemiology and Infection.

[ref25] (2019). Gender and time delays in diagnosis of pulmonary tuberculosis: a cross-sectional study from China. Epidemiology and Infection.

[ref26] (2019). Trend in risk of delay in diagnosis of new pulmonary tuberculosis in Northwest China from 2008 to 2017. BMC Infectious Diseases.

[ref27] (2018). Patient, healthcare system and total delay in tuberculosis diagnosis and treatment among Serbian population. Acta Clinica Croatica.

[ref28] (2017). Diagnostic delay and associated factors among patients with pulmonary tuberculosis in Kerala. Journal of Family Medicine and Primary Care.

[ref29] (2018). Factors associated with treatment delay among newly diagnosed tuberculosis patients in Dessie city and surroundings, Northern Central Ethiopia: a cross-sectional study. BMC Public Health.

[ref30] (2018). Healthseeking behaviour and treatment delay in patients with pulmonary tuberculosis in Switzerland: some slip through the net. Swiss Medical Weekly.

